# Kaempferide in *Alpinia officinarum* Hance Exerts Anti‐Hepatocellular Carcinoma Activity by Inhibiting Mitophagy Through Downregulation of BNIP3L

**DOI:** 10.1111/jcmm.71249

**Published:** 2026-07-14

**Authors:** Lu Lu, Changxian Li, Cunzhen Jiang, Yuan Zhao, Yinghong Zhong, Jicheng Hu, Zhe Wang, Mingyan Zhou, Jian Xu, Xiangcheng Li

**Affiliations:** ^1^ Hepatobiliary Center The First Affiliated Hospital of Nanjing Medical University Nanjing China; ^2^ Hepatobiliary and Liver Transplantation Department of Hainan Digestive Disease Center The Second Affiliated Hospital of Hainan Medical University Haikou China; ^3^ Institute of Clinical Medicine The Second Affiliated Hospital of Hainan Medical University Haikou China; ^4^ Key Laboratory of Emergency and Trauma of Ministry of Education The First Affiliated Hospital of Hainan Medical University Haikou China

**Keywords:** *Alpinia officinarum* Hance, BNIP3L, hepatocellular carcinoma, kaempferide, mitophagy

## Abstract

Hepatocellular carcinoma (HCC), the most common primary malignant tumour of the liver, is notorious for its high mortality rate. *Alpinia officinarum* Hance ( *A. officinarum* ) is a perennial medicinal herb used for the management of abdominal pain, vomiting, and gastrointestinal tumours, and is widely applied as a dietary intervention by the Li ethnic group in Hainan, China. Kaempferide, a bioactive flavonoid isolated from this herb, exhibits promising antitumor properties; however, its precise mechanism against HCC remains incompletely understood. In this study, we demonstrated that kaempferide could inhibit proliferation and migration of HCC cells, induce G0/G1 phase cell arrest and promote the accumulation of ROS, while suppressing xenograft tumour growth in nude mice. Further investigations revealed that kaempferide targeted BNIP3L and suppressed mitophagy, evidenced by elevated P62 levels and reduced LC3‐II levels. In summary, kaempferide exerts anti‐HCC effects by inhibiting mitophagy via the downregulation of BNIP3L, suggesting its potential role as a therapeutic candidate for HCC.

**Abbreviations**
AOE: A officinarum extract
A.officinarum: Alpinia officinarum Hance
Beclin1: Moesin‐like BCL2‐interacting protein
BNIP3: BCL2 interacting protein 3
BNIP3L: BCL2 interacting protein 3 like
CCK8: Cell counting kit‐8
EdU: 5‐ethyl‐2′‐deoxyuridine
GO: Gene ontology
HCC: Hepatocellular carcinoma
HE: Haematoxylin–eosin
HIF‐1α: Hypoxia‐inducible factor 1 subunit alpha
IHC: Immunohistochemistry
KEGG: Kyoto Encyclopedia of Genes and Genome
LC3‐II: Microtubule‐associated protein 1 light chain 3‐II
P62: Ubiquitin‐binding protein p62
qRT‐PCR: Quantitative reverse transcription‐PCR
TCM: Traditional Chinese medicine
UPLC‐MS/MS: Ultra‐high performance liquid chromatography‐mass spectrometry/mass spectrometry
WB: Western blot

## 1 Introduction

Liver cancer constitutes roughly 850,000 incidences and 800,000 fatalities yearly, positioned as the secondary principal contributor to oncological demise. HCC represents the predominant variant of primary liver cancer, comprising 85%–90% of instances [1]. Hepatectomy and liver transplantation are the optimal curative treatments for curing liver cancer but their success is hampered by a high rate of recurrence, limited liver donors, and a poor prognosis. In addition, since a majority of patients are identified during a progressed phase, they forgo the chance for surgery and can only receive drug therapy [2]. For patients with unresectable HCC, the multi‐kinase inhibitor sorafenib is a first‐line oral therapeutic option. Although it yields certain clinical efficacy, its clinical use is limited by adverse effects and the development of drug resistance [3]. Traditional Chinese Medicine (TCM), a treasure of China, has been validated as beneficial in ameliorating clinical manifestations and enhancing well‐being among individuals suffering from liver cancer, reducing recurrence, controlling disease progression, and improving drug tolerance [4].

For centuries, TCM has been widely employed in the treatment of a broad range of diseases owing to its favourable safety, efficacy, and cost‐effectiveness. In China, *A. officinarum* is the prominent traditional Li medicine with many clinical applications [5]. To date, chemical entities have been isolated from *A. officinarum* , predominantly comprising mostly the active ingredients, such as flavonoids, diarylheptanoids, phenylpropanoids, glycosides, and volatile oils [6]. With the rapid advancement in pharmacology, researchers have scientifically validated multiple pharmacological effects of *A. officinarum* , including treating metabolic disorders [7], anti‐ulcer properties [8], anti‐inflammatory and analgesic effects [9], antioxidant activity [10], anti‐tumour effects [11, 12], inhibition of liver injury [13], antibacterial properties [14], and hypoglycemic effects [15]. Our research team has already looked into the partial pharmacological mechanisms of diarylheptanoids and the flavonoid kaempferol originating from *A. officinarum* using in vivo and In vitro investigations for liver cancer and diabetes [7, 11, 12, 16].

Kaempferide (KF; 3,5,7‐trihydroxy‐4′‐methoxyflavone) is a bioactive flavonoid compound that is contained in *A. officinarum* . In terms of molecular structure, kaempferide is an O‐methyl derivative belonging to the compound kaempferol with anticancer activity against HCC [17]. The fundamental progenitor flavonoid scaffold of 2‐phenyl‐chromone (A) is a flavonoid for both kaempferide and kaempferol. The substitution of a phenolic hydroxyl moiety at the fourth site of the B‐ring in kaempferol versus a methoxy group in kaempferide. Kaempferide and kaempferol demonstrate an array of therapeutic properties, encompassing antineoplastic, counter‐inflammatory, antioxidative, antidiabetic, anti‐adiposity, and neurological preservation effects [18, 19, 20, 21]. However, in the crucial characteristic of pharmacokinetics, kaempferide outperforms other flavonoids such as kaempferol [22]. Gopika Chandrababu et al. have established that kaempferide triggers HCC cellular demise In vitro. In mice, kaempferide reduced the volume of the tumour and increased caspase‐9. Moreover, it was shown to downregulate transforming growth factor‐β1 (TGF‐β1) [17]. This observation attests to the antineoplastic promise of kaempferide, although the therapeutic pathways fundamental to the anti‐HCC impacts of galangal‐derived kaempferide necessitate additional examination.

This study utilized multiple HCC cell lines as experimental models and combined a subcutaneous xenograft tumour model in nude mice to investigate the molecular mechanism of kaempferide. The results yield scientific proof of the practicable use of kaempferide from *A. officinarum* in HCC treatment but also provide innovative methods to develop small molecule drugs against hepatocellular carcinoma.

## 2 Materials and Methods

### 2.1 Chemicals and Reagents

The standard drugs including kaempferide were supplied by Chengdu Pufeide Reference Standard Technology Co. Ltd., with a purity exceeding 98%. DMEM medium (1X) (Cat. No. C11995500BT) was acquired from GIBCO. Fetal bovine serum (Cat. No. FSP500) was obtained from ExCell Bio. CCK8 assay kit (Cat. No. K1018) was acquired from APExBIO. The Apoptosis analysis kit (40302ES50), Hifair III 1st Strand cDNA Synthesis SuperMix for qPCR (gDNA digester plus, Cat. No. 11141ES60) and cell cycle kit (Cat. No. C1052) were procured from Yeasen Biotechnology (Shanghai) Co. Ltd. The Eastep Super Total RNA Extraction Kit (Cat. No. LS1040) was purchased from Promega Biotechnology (Shanghai) Co. Ltd. The BeyoClickTM EdU‐555 Cell Proliferation Detection Kit (Cat. No. C0075S), Haematoxylin and Eosin (H&E) Staining Kit (Cat. No. C0105S‐1,2), MDC staining assay kit for cellular autophagy detection (Cat. No. C3018S) and Reactive Oxygen Species Assay Kit (Cat. No. S0033S) were supplied by Beyotime Biotechnology Co. Ltd. Human PCR primer sequences for HIF‐1α, Beclin1, BNIP3, BNIP3L, LC3‐II, p62, and GAPDH were acquired from Guangzhou Tianyi Huiyuan Biotechnology Co. Ltd. HIF‐1α antibody (AF1009), Beclin 1 antibody (AF5128), BNIP3L antibody (DF8163), BNIP3 antibody (DF8188), P62 antibody (AF5384), LC3‐II antibody (AF5402), Ki67 antibody (AF0198), GAPDH (AF7021),and β‐actin (AF7018) were purchased from AFFINITY. Lentiviral vectors carrying BNIP3L overexpression construct and mRFP‐GFP‐LC3 dual fluorescence reporter were constructed and purchased from Shanghai GeneChem Co. Ltd.

### 2.2 Identification of Active Compounds in *A. officinarum* Extract by UPLC‐MS/MS

The *A. officinarum* extract (AOE) sample is stored in the Medicinal Chemistry Laboratory of Hainan Medical University (voucher number: HY2013012237) [23]. The active compounds in AOE were identified using the HY5600 liquid chromatography–tandem mass spectrometry system (Shenzhen Heyue Biotechnology Co. Ltd.). The analysis techniques and parameters are listed below: A Phenomenex Kinetex C18 column (2.1 × 100 mm, 1.7 μm) was utilized; mobile phase A comprised 0.25 mmol/L ammonium fluoride water‐based solution, and mobile phase B constituted 0.5 mmol/L ammonium fluoride methanol‐based solution. The injection volume was 5 μL for each sample. Three components (DPHC, DPHB, DPHA) were tested in active ion mode, while three other components (kaempferide, kaempferol, galangin) were tested in negative ion mode, with separate scanning for positive and negative ions. The analysis time for each sample was 6.5 min. Gradient elution was applied.

### 2.3 Molecular Docking

The molecular structure of kaempferide was retrieved from the PubChem database, whereas the target protein structure was extracted from UniProt database. The water molecules and ligands of the receptor protein were eliminated via PYMOL 2.3.4 software. Using AutoDockTools, the receptor protein was modified by adding hydrogen atoms and balancing the charges. Molecular docking of the receptor protein with the small molecule ligand was performed employing AutoDock Vina 1.1.2. The docking process was visualized using PyMOL Software.

### 2.4 Cell Culture and Transfection

The HepG2, HuH7 and HCCLM3 HCC cell lines were supplied by Professor Junqing Zhang from the Department of Pharmacy, Hainan Medical University. The cells were cultured in medium containing 10% fetal bovine serum, 100 U/mL penicillin and 100 μg/mL streptomycin. The sequence for the overexpression of BNIP3L was purchased from Shanghai GeneChem Co. Ltd. Plasmids carrying the BNIP3L overexpression sequence were transfected into HCCLM3 cells using PEI 40 K transfection reagent (Cyagen Biosciences, Wuhan, China) following the manufacturer's instructions. Upon successful transfection, HCC cells were treated with gradient concentrations of kaempferide for 24 h. Finally, we gathered cell genes and protein molecules for subsequent examination.

### 2.5 Cell Viability Assay (CCK8)

HCC cells were then plated into 96‐well plates at a density of 3000 cells per well. The plates were incubated at 37°C and 5% CO_2_. The following day, cells were treated with different concentrations of kaempferide for 24 h. After removing the culture medium, 100 μL of CCK8 reagent was added to each well and the plate was further incubated for one hour. Absorbance was measured using a microplate reader at a wavelength of 450 nm.

### 2.6 Assessment of Autophagic Flux

HCC cells were infected with an mRFP‐GFP‐LC3 tandem fluorescent lentivirus (Shanghai GeneChem) at an optimal MOI for 48 h, followed by treatment with kaempferide,CCCP or CQ for 24 h. Fluorescent images were acquired using an FV3000 laser scanning confocal microscope (Olympus, Tokyo, Japan). Autophagic flux was evaluated by quantifying the number of autophagosomes (yellow puncta) and autolysosomes (red puncta) per cell from randomly selected fields of view.

### 2.7 EdU Detection

HCC cells were treated with different concentrations of kaempferide for two hours, followed by the addition of 2 × EdU working solution. The absorbance was measured at a wavelength of 450 nm using a microplate reader. Then, 4% paraformaldehyde was administered to the wells and cells were stabilized for 15 min. Afterwards, cells were treated with 0.25% Triton‐X 100 solution for 15 min to permeate the cell membranes. Then added the supplemented click reaction additive solution and incubated for 30 min, followed by Hoechst staining for 10 min and then checked under a fluorescence microscope.

### 2.8 Scratch Test

HCC cells were plated into a 6‐well plate and cultured for 24 h. Use a pipette tip to make a straight line in each well and wash away the detached cells with PBS. Then treat the cells in the 6‐well plates with different concentrations of kaempferide. Wound healing processes in the respective wells were observed under an optical microscope, and photo data were analysed and evaluated using Image J software.

### 2.9 Flow Cytometry

For the apoptosis detection assay, Annexin V‐FITC and propidium iodide (PI) staining solutions were added to the cells and incubated in dark for 10–15 min. Cell apoptosis rates were then determined by flow cytometry. For cell cycle assay, cells were fixed with 80% ethanol, followed by adding PI and incubation at 37°C in the dark for 30 min. Finally, cell cycle distribution was analysed by flow cytometry.

### 2.10 ROS Determination

HCC cells seeded in wells were treated with various concentrations of kaempferide. Following 24‐h incubation at 37°C in a 5% CO_2_ incubator, the medium was removed, and cells were gently washed three times with extracellular solution. Cells were then incubated with 10 μM DCFH‐DA (final concentration) for 30 min at 37°C in the dark. After incubation, DCFH‐DA was discarded, and cells were rewashed to remove uninternalized probe. ROS levels were quantified by flow cytometry, and their cellular localization was visualized by a confocal laser scanning microscope.

### 2.11 RNA‐Seq Analysis

RNA sequencing (RNA‐seq) and data analysis were carried out on the group of HCC cells that was exposed to different concentrations of kaempferide and the untreated HCC cell group. First, total RNA of all cell samples was extracted. The purity, concentration and integrity of the samples were measured. To begin with, for all the samples the paired‐end mode was used to carry out high‐throughput sequencing on the Illumina HiSeq. The DESeq2 application was employed to detect differentially expressed genes among cohorts employing the differential expression thresholds of |log2 FC| ≥ 1, *p* − ≤ 0.05. Gene Ontology functional examination utilizing the TopGO application and Kyoto Encyclopedia of Genes and Genomes (KEGG) repository pathway investigation was performed on the target gene collection, correspondingly. Finally,a total of 2433 significantly upregulated genes and 3355 significantly downregulated genes were identified in the RNA‐seq profile.

### 2.12 Autophagy Staining Assay (MDC Method)

To make the 1 × Assay Buffer, firstly dilute the 10 × Assay Buffer with sterile deionized water at a ratio of 1:10. Afterwards, use the 1000 × MDC stock solution to freshly prepare a 1 × MDC staining working solution by diluting 1:1000 in 1 × Assay Buffer (all operations under light protected conditions). During the logarithmic growth phase, seed HCCLM3 cells and culture them until they reach 60%–70%. Divide cells into control and kaempferide treatment groups. Discard the culture medium and add MDC working solution, then incubate cells for 30 min at 37°C in the dark. After washing with 1 × Assay Buffer three times, the cells were observed using a fluorescence microscope; autophagosomes should appear as green fluorescent spots. For quantification, the fluorescence strength may be assessed utilizing a microplate reader or flow cytometer.

### 2.13 Transmission Electron Microscope (TEM)

When the endpoint of the cell treatment is reached, wash the cells 2–3 times with pre‐cooled 1 × PBS, digest with trypsin and neutralize the digestion with complete DMEM medium to collect into a 5 mL centrifuge tube, followed by centrifuging at 600 × g for 10 min at 4°C. The supernatant was discarded and the pellet was gently resuspended in 1 × PBS. Then the tube was centrifuged at 600 × g, 4°C for 10 min. The upper liquid phase was discarded and the fixative solution was added into the tube without dispersing the cells. Fix at 4°C for 2 h. After fixation, rinse samples 3 times with electron microscopy buffer and send to Hainan Medical University Electron Microscopy Building for sectioning. Monitor the maturity of mitochondria and mitophagy using TEM.

### 2.14 HCCLM3 Cell Xenograft Tumour Model

4‐week‐old male BALB/c nude mice were acclimated for 2 weeks before subcutaneous implantation of HCCLM3 cell suspension (5 × 10^6^ cells) into the right axillary region. When the tumour volume reached approximately 100 mm^3^, the mice were divided into five groups randomly (*n* = 10 per group). Kaempferide was administered at low, medium, and high doses, while the positive control group received sorafenib. All groups were administered orally once daily for 14 days consecutively. The control group received an equivalent volume of sodium carboxymethyl cellulose solution. Tumour volume and body weight were measured every 2 days. At the end of the treatment period, serum samples were collected for biochemical analysis, and tumours with key organs were collected for calculating organ coefficients and conducting histological examinations. All animal experiments were conducted in accordance with the Animal Ethics Guidelines of Hainan Medical University (Approval No: HYLL‐2024‐807).

### 2.15 H&E Staining

Liver, spleen, and kidneys from nude mice were fixed in 4% paraformaldehyde, followed by paraffin embedding and sectioning. The paraffin sections were deparaffinized and rehydrated through a graded ethanol series, then stained with haematoxylin and eosin. Microscopic observation was performed to evaluate the in vivo toxic effects of kaempferide on nude mice. For HE staining and IHC observation, a 3DHISTECH Pannoramic MIDI digital slide scanner was used for image acquisition and analysis.

### 2.16 IHC Determination

After dewaxing and rehydration, the paraffin sections were subjected to antigen retrieval, followed by blocking of endogenous peroxidase activity with hydrogen peroxide to minimize nonspecific staining. The sections were then incubated with the primary antibody overnight at 4°C, washed with PBS, and treated with an HRP‐conjugated secondary antibody. After three PBS washes, the sections were stained with diaminobenzidine (DAB) for colour development, counterstained with haematoxylin, and observed under a microscope for image acquisition.

### 2.17 qRT‐PCR

Total RNA was extracted using an RNA extraction kit, and reverse transcribed to cDNA. Real‐time quantitative reverse transcription qRT‐PCR was performed in a thermal cycler employing SYBR Green dye. Transcript abundance of the target genes' mRNA was standardized relative to the expression level of GAPDH or *β*‐actin as a reference internal control, and the results were evaluated using the 2^ΔΔCt^ method. Primer sequences for qRT‐PCR were retrieved from NCBI (https://www.ncbi.nlm.nih.gov/) and are shown in Table 1.

**TABLE 1:** Primer sequences for qRT‐PCR.

Gene	Forward (5′‐3′)	Reverse (5′‐3′)
HIF‐1α	TGAAGTGTACCCTAACTAGCCG	TCACAAATCAGCACCAAGC
Beclin1	ACCAGATGCGTTATGCCC	CGACCCAGCCTGAAGTTAT
BNIP3	ACCTCCACCAGCACCTTT	GGAACGCAGCATTTACAGA
BNIP3L	CCAGTAGACCCGAAAACATT	GCTCAGTCGCTTTCCAATA
LC3‐II	GTTACGGAAAGCAGCAGTG	GGAAGGCAGAAGGGAGTGT
p62	ACCACTTTTGCCCACCTC	TGCACCCTAACCCCTGAT
GAPDH	CCTTCCGTGTCCCCACT	GCCTGCTTCACCACCTTC

### 2.18 Western Blot

Cells and tumour tissue homogenates were lysed using RIPA lysis buffer containing 1% protease inhibitor. The supernatants were collected to determine protein concentrations, and then the protein samples were mixed with loading buffer. The samples were separated by SDS‐PAGE and were transferred onto PVDF membranes. Subsequently, the membranes were blocked with 5% skim milk for 1 h at room temperature, followed by incubation with primary antibodies overnight at 4°C. The next day, the membranes were washed with TBST (containing 1% Tween‐20) three times and then incubated with HRP‐conjugated secondary antibodies for 1 h at room temperature. After three washes, the chemiluminescence signal was detected using an ECL reagent and captured by the Tanon imager. The grayscale values were analysed using ImageJ software.

### 2.19 Data Analysis

All experiments were performed in triplicate. All data are presented as mean ± standard deviation. Two‐tailed paired Student's *t*‐test was used for comparisons between two groups, while one‐way ANOVA was applied for intergroup comparisons. Data processing was implemented using GraphPad Prism 8 software. A *p* > 0.05 was considered statistically significant.

## 3 Results

### 3.1 Analysis of the Active Ingredients in *A. officinarum* Extract (AOE)

**FIGURE 1:**
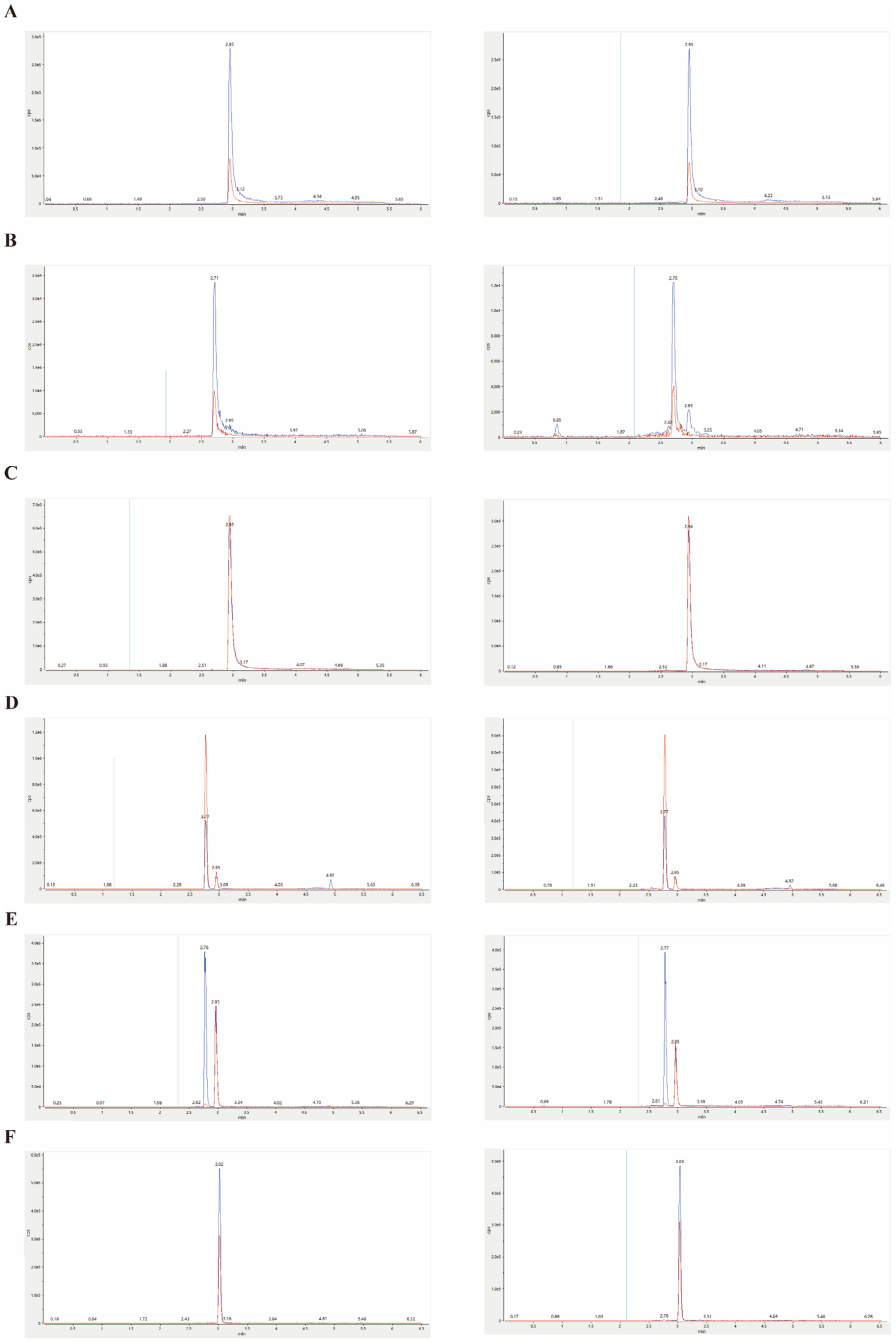
Chromatograms of 6 active components in AOE and reference standards (left: Chromatogram of reference standards, right: Chromatogram of 6 active components in AOE). (A) Kaempferide, (B) Kaempferol, (C) Galangin, (D) DPHA, (E) DPHB, (F) DPHC.

AOE was systematically analysed utilizing ultra‐high performance liquid chromatography–tandem mass spectrometry (UPLC‐MS/MS). Six active ingredients were targeted and identified in AOE, namely kaempferide, kaempferol, galangin, DPHA (5‐hydroxy‐1‐phenyl‐7‐(4‐hydroxy‐3‐methoxyphenyl)‐3‐heptanone), DPHB (7‐(4‐hydroxy‐3‐methoxyphenyl)‐1‐phenylhept‐4‐en‐3‐one), and DPHC ((R)‐5‐hydroxy‐1,7‐diphenyl‐3‐heptanone). The chromatogram of the active component is shown in Figure 1. The kaempferide standard and the active component of kaempferide in AOE are basically identical.

### 3.2 Kaempferide Can Inhibit the Proliferation and Migration of HCC Cells

**FIGURE 2:**
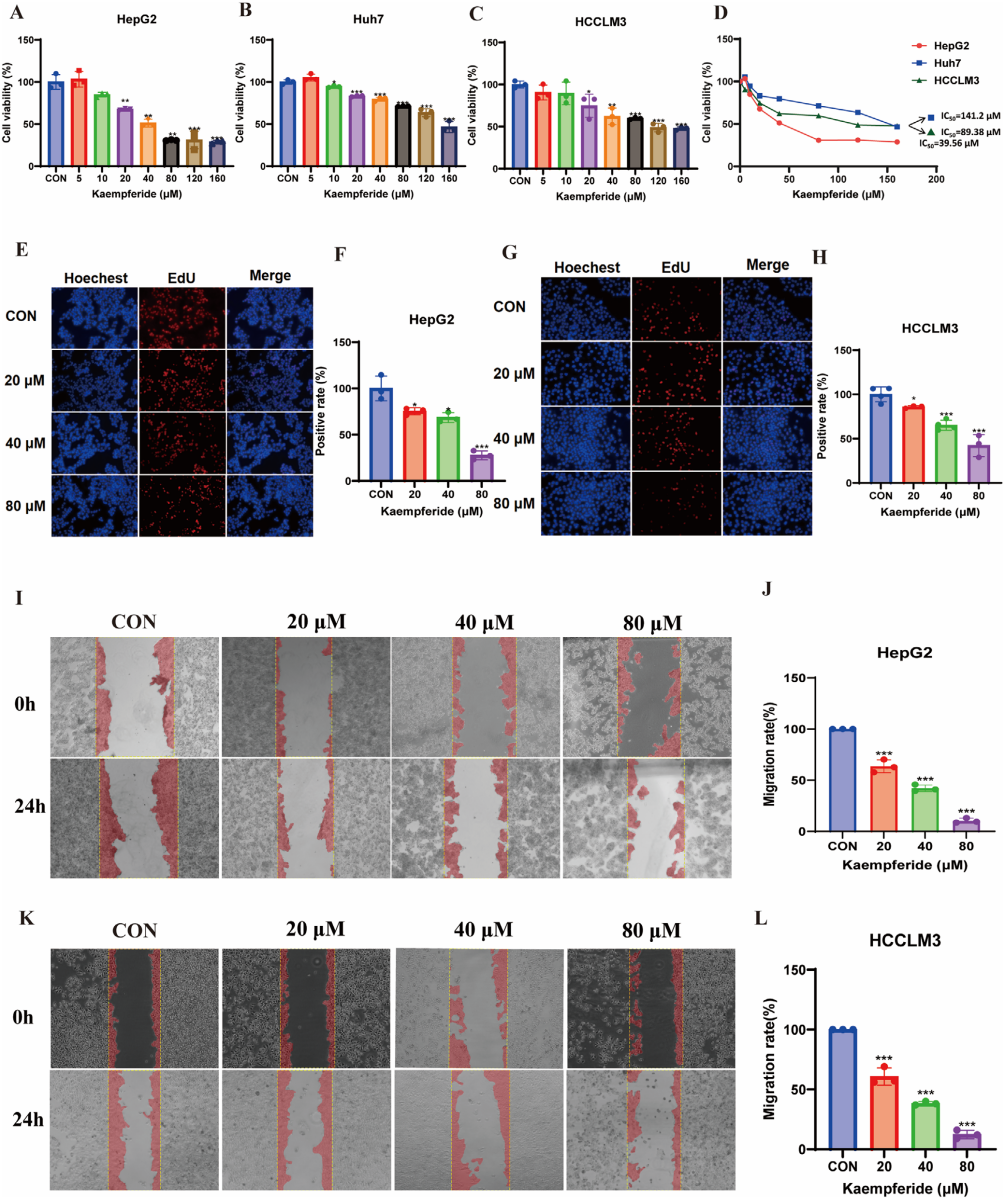
Inhibitory effects of kaempferide on proliferation and migration of HCC cell lines. (A‐C) The viability of HepG2, Huh7, and HCCLM3 cells treated with kaempferide was measured using CCK8 assay. (D) The IC50 values of HCC cell lines were determined individually.(E‐H) The proliferative capacity of HCC cells treated with kaempferide was determined by EdU assay. Red fluorescence and blue fluorescence indicated EdU‐positive cells and Hoechst‐positive cells, respectively. (I‐L) Scratch assay was performed to evaluate the effect of kaempferide on the migratory capability of HCC cells. Data were presented as mean ± standard (*n* = 3). *p < 0.05, **p < 0.01, ***p < 0.001 vs. the CON group.

Based on the data obtained from CCK8 results, the growth of the HuH7, HepG2 and HCCLM3 cell lines was significantly inhibited from 20 μM onwards (*p* < 0.05, Figure 2A–C), indicating the proliferation inhibition profile of Kaempferide for HCC cells. IC50 value of the Kaempferide in HepG2, HCCLM3 and HuH7 cells were 43.69 μM, 136.1 μM and 150 μM respectively (Figure 2D). The EdU outcomes revealed that kaempferide at concentrations of 20 μM, 40 μM, and 80 μM suppressed the proliferation of HCC cell lines in a concentration‐dependent fashion (*p* < 0.05, Figure 2E–H). This was succeeded by the scratch examination, wherein we noted that kaempferide restrained the HCC cellular migration in a concentration‐dependent fashion (*p* < 0.001, Figure 2I–L).

### 3.3 Kaempferide May Affect Mitophagy in HCC via Downregulating BNIP3L

**FIGURE 3:**
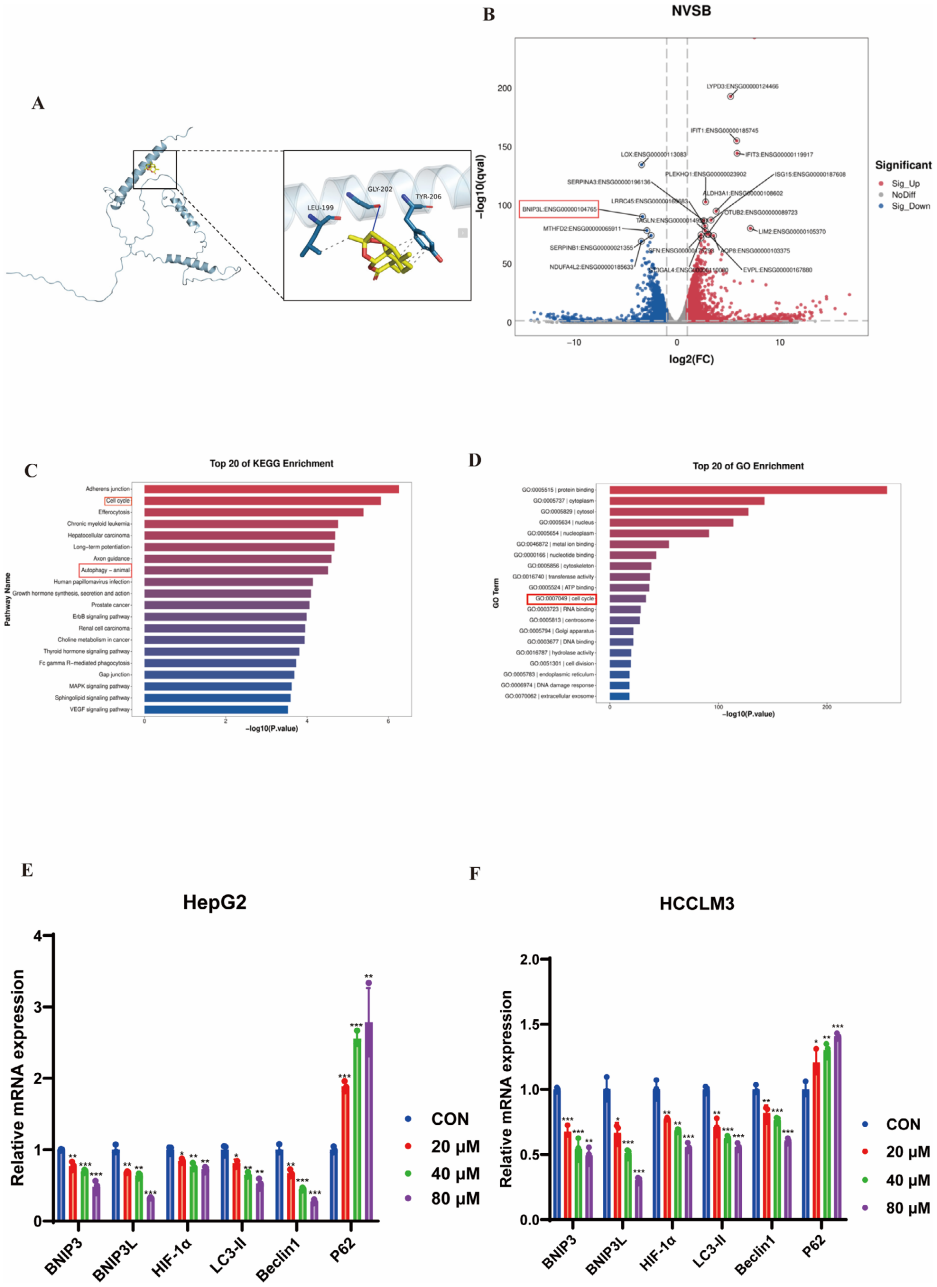
Kaempferide may affect mitophagy in HCC cells. (A) Kaempferide can directly bind to the target protein BNIP3L through its specific spatial conformation. (B) Volcano plot of gene expression. Elevated genes are depicted in crimson on the right, whereas reduced genes are presented in azure on the left. (C) GO assessment. (D) KEGG assessment. (E,F) Transcription quantities of BNIP3L and mitophagy‐associated genes within HepG2 and HCCLM3 cells following exposure to varying concentrations of kaempferide. Information is exhibited as mean ± standard (*n* = 3). *p < 0.05, **p < 0.01, ***p < 0.001 vs. the CON group.

The kaempferide treated HCC cells were subjected to transcriptome sequencing along with a control group of cells to get the complete picture on benefits of kaempferide in HCC cells on a genetic level. The volcano plot (Figure 3B) was used to visualize the gene expression changes. GO and KEGG analyses revealed significant alterations in HCC cells' autophagy pathways and cell cycle upon kaempferide treatment (Figure 3C,D). Among the differentially expressed genes depicted in the volcano plot (Figure 3B), the mitophagy gene BNIP3L was markedly downregulated. According to Zhang and Ney [24], the expression of BNIP3L‐dependent mitophagy‐related genes BNIP3, HIF‐1α, Beclin1, and LC3‐II is significantly upregulated during mitophagy. The treatment of Kaempferide caused downregulation of the indicated genes via sequencing. Molecular docking analysis showed that kaempferide exhibited the ability to bind to BNIP3L in a certain spatial conformation, showing a binding energy of −5.1 kcal/mol (Figure 3A). This binding motif reveals the molecular mechanism through which kaempferide regulates the BNIP3L‐related signalling pathway and exerts its anti‐HCC activity. We utilized qRT‐PCR to confirm the transcription of BNIP3L‐dependent mitophagy‐associated genes within HCC cells (Figure 3E,F). The qRT‐PCR results showed that the transcription of HIF‐1α, BNIP3L, BNIP3, Beclin1, and LC3‐II was markedly reduced post kaempferide administration (*p* < 0.05). Conversely, the transcription of P62 was substantially elevated after kaempferide administration (*p* < 0.05).

### 3.4 Kaempferide Induces G0/G1 Phase Arrest in Hepatocellular Carcinoma Cells and Inhibits Their Mitophagy Process

**FIGURE 4:**
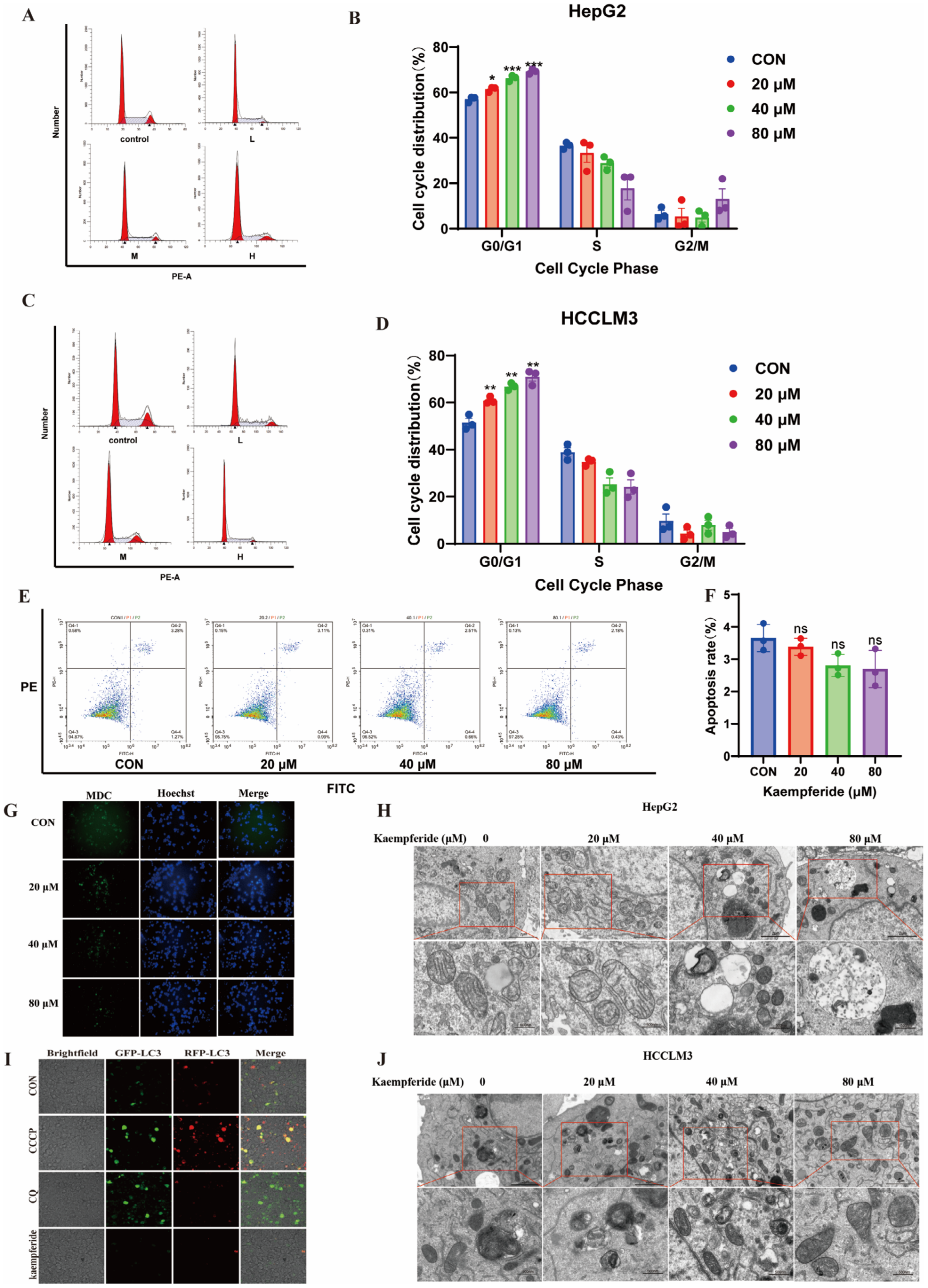
Kaempferide induces cell cycle arrest at G0/G1 phase and inhibits mitophagy in HCC cells. (A–D) Effects of kaempferide on cell cycle distribution in HepG2 and HCCLM3 cells. (E, F) Kaempferide did not affect the apoptosis process in HepG2 cells. (G) MDC assay showed decreased autophagosomes in HCCLM3 cells after kaempferide treatment. (I) The assessment of autophagic flux revealed that kaempferide blocks the mitophagic flux in HCCLM3 cells. (H, J) Representative transmission electron micrographs of HepG2 and HCCLM3 cells after kaempferide treatment. Scale bars, 2 μm. Data are presented as mean ± standard deviation (*n* = 3/group). *p < 0.05, **p < 0.01, ***p < 0.001 vs. the CON group.

We employed flow cytometry to analyse the effect of kaempferide on the programmed cell death and cell cycle progression in HCC cells. The results indicated that kaempferide significantly induced G0/G1 phase cell cycle arrest in HCC cells (*p* < 0.05) (Figure 4 A‐D). However, it exerted no significant effect on apoptosis (*p* > 0.05) (Figure 4 E‐F). According to the results of the qRT‐PCR (Figure 3E,F), kaempferide significantly downregulated the expression level of BNIP3L in HCC cells. Cellular autophagy assay results showed that kaempferide dose‐dependently inhibited mitophagy (Figure 4G). Autophagic flux assessment further confirmed that kaempferide blocks mitophagic flux in HCC cells (Figure 4I). Furthermore, transmission electron microscopy revealed that kaempferide treatment led to mitochondrial injury and the accumulation of damaged mitochondria in HCC cells (Figure 4H,J). It was further confirmed that kaempferide inhibits mitophagy in HCC cells.

### 3.5 Kaempferide Exerts Anticancer Effects by Downregulating BNIP3L to Suppress Mitophagy in HCC

**FIGURE 5:**
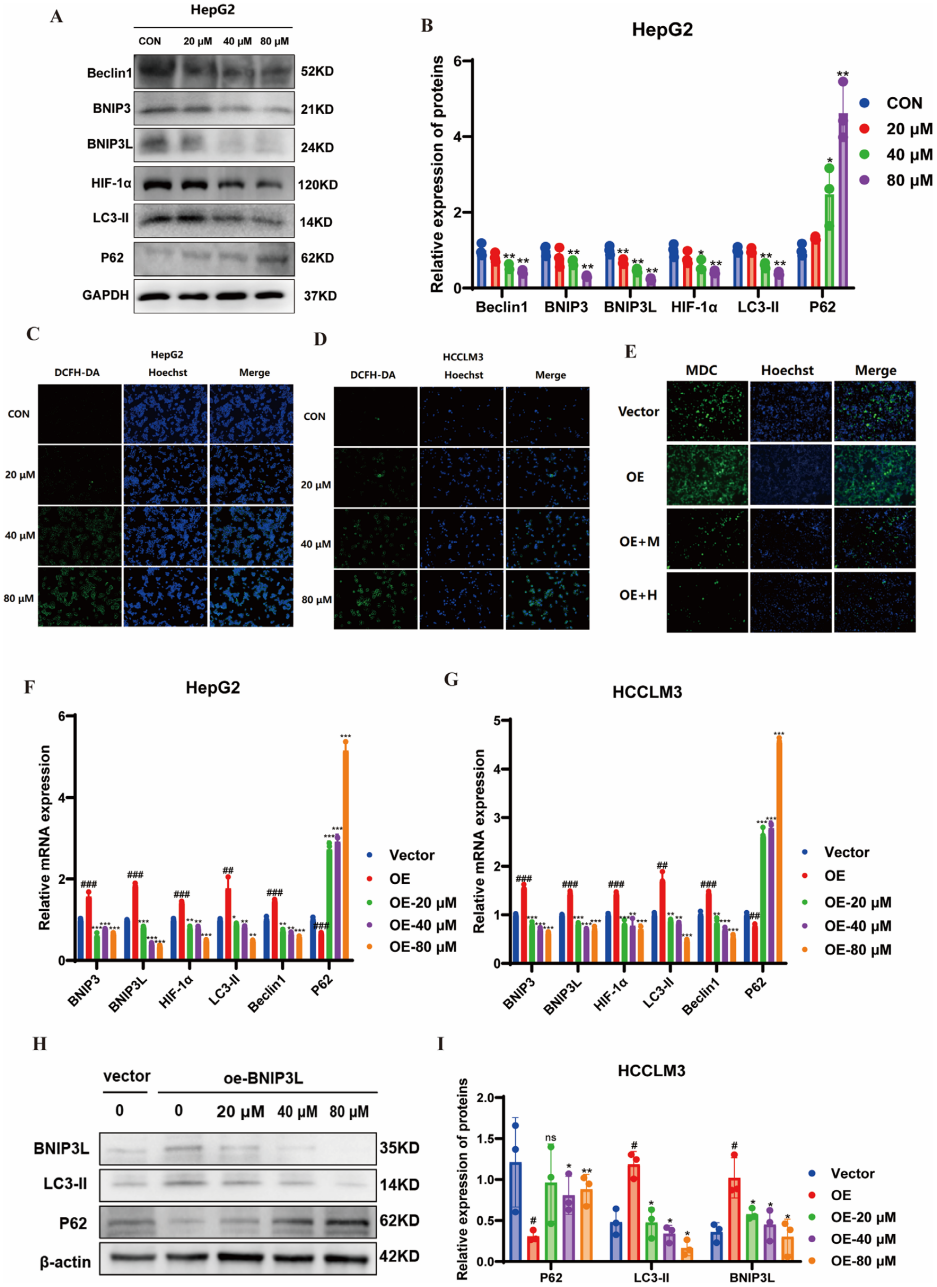
Kaempferide inhibits HCC cell mitophagy and exerts anticancer activity by downregulating BNIP3L. (A‐B) Changes in target protein expression in HepG2 cells after kaempferide treatment. (C‐D) ROS levels were dramatically raised in HepG2 and HCCLM3 cells after kaempferide treatment. (E) MDC staining revealed that kaempferide reversed the BNIP3L‐induced increase of autophagosomes in HCCLM3 cells. (F‐G) Expression levels of key target genes. (H‐I) Analysis results of key target protein expression levels in BNIP3L‐overexpressing cells after kaempferide administration. #*p* < 0.05, #*p* < 0.01, #*p* < 0.001 vs. the Vector group. Data are presented as mean ± standard (*n* = 3). *p < 0.05, **p < 0.01, ***p < 0.001 vs. the BNIP3L group (overexpression of BNIP3L). Vector: Negative‐control of BNIP3L. OE: Overexpression of BNIP3L. OE‐20 μM: Overexpression of BNIP3L and treatment with 20 μM kaempferide. OE‐40 μM (OE + M): Overexpression of BNIP3L and treatment with 40 μM kaempferide. OE‐80 μM (OE + H): Overexpression of BNIP3L and treatment with 80 μM kaempferide.

The reduction of LC3‐II and increase of P62 downstream of BNIP3L signify the retardation in mitophagy advancement [25]. According to Ng and colleagues [26]. Cancer patients may benefit from mitophagy inhibition. Absence of HIF‐1α, BNIP3 and/or BNIP3L manifestation can trigger cellular demise, but elimination of Beclin1 intensifies cellular demise under oxygen‐deprived settings [27]. The result of this research sequencing concurs with the data of our exploration and the qRT‐PCR results after kaempferide treatment in HCC cells (Figure 3E,F). According to WB analysis, HepG2 cells experienced expression changes of relevant proteins (Figure 5A,B). Research shows that disrupted mitochondria raise intracellular ROS levels, leading to cell death [28]. For this purpose, ROS content in HepG2 and HCCLM3 cells was assessed after kaempferide treatment. According to the results, kaempferide could raise ROS levels in HCC cells (Figure 5C,D). MDC staining revealed that BNIP3L overexpression increased the number of autophagosomes in HCCLM3 cells, while kaempferide treatment reversed this trend (Figure 5E). According to the qRT‐PCR and WB experimental results, BNIP3L overexpression downregulated P62 expression and upregulated LC3‐II expression in HCCLM3 cells, indicating the activation of the mitophagy pathway. Rescue experiment further confirmed that kaempferide treatment reversed the above phenomenon (*p* < 0.05) (Figure 5F–I). In conclusion, kaempferide‐induced inhibition of mitophagy in HCC cells occurs via downregulation of BNIP3L expression.

### 3.6 Kaempferide Inhibits HCC Tumour Growth In Vivo

**FIGURE 6:**
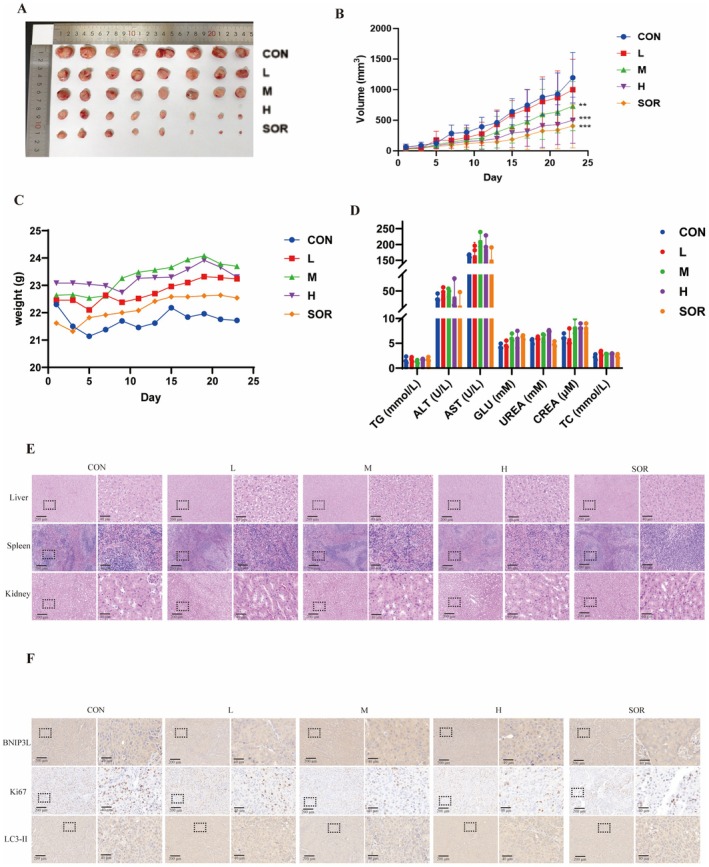
Effectiveness and initial security appraisal of kaempferide in managing HCC in vivo. (A) Images of neoplasm specimens from every test cohort. (B, C) Measurement of tumour size and creature mass at designated intervals (*n* = 8/group). (D) Manifestation quantities of biochemical indicators in creature serum were appraised (*n* = 4). (E) HE staining examination of organ structures from every cohort. (F) IHC examination of BNIP3L, LC3‐II and Ki67. Information is displayed as average ± standard. *p < 0.05, **p < 0.01, ***p < 0.001 vs. the CON group. Low dose group (L Group): 10 mg/kg kaempferide orally administered; Medium dose group (M Group): 20 mg/kg kaempferide orally administered; High dose group (H Group): 40 mg/kg kaempferide orally administered; Sorafenib group (SOR Group): 20 mg/kg Sorafenib orally administered.

We established an HCCLM3 cell xenograft tumour model for studying the in vivo therapeutic effect of kaempferide on HCC. The mice were then given kaempferide (sodium carboxymethyl cellulose) orally via a cannula. Compared with the control group (CON group), kaempferide significantly inhibited tumour growth (*p* < 0.01) (Figure 6A,B), without causing any significant change in body weight (*p* > 0.05) (Figure 6C). We determined serum transaminase (ALT and AST) levels of mice to evaluate the possible hepatotoxicity of kaempferide. Investigation revealed that no disparity in transaminase concentrations was detected among the kaempferide group and control group. This indicates that kaempferide does not show detectable hepatotoxicity (*p* > 0.05) (Figure 6D). Likewise, UREA and CREA levels in the kaempferide group did not significantly change compared to control, indicating that kaempferide did not exert a detectable nephrotoxic effect (*p* > 0.05) (Figure 6D). Furthermore, there were no notable alterations in blood glucose (GLU) or lipid profiles (total cholesterol, TC; triglycerides, TG) of nude mice (*p* > 0.05) (Figure 6D). The organ tissue of all groups showed normal morphology, uniform staining and regular cellular arrangement as seen in H& E Application (Figure 6E). IHC results showed that, compared to the CON group, the expression levels of BNIP3L, LC3‐IIand Ki67 were reduced in the kaempferide‐treated group (Figure 6F). These results all indicate that kaempferide inhibits in vivo tumour growth in the absence of hepatotoxicity, splenotoxicity or nephrotoxicity. Thus, this provides significant support for further research into the mechanistic basis of kaempferide as an anticancer drug and its effect on human health.

### 3.7 Kaempferide Improves HCC by Modulating BNIP3L Expression Levels In Vivo

**FIGURE 7:**
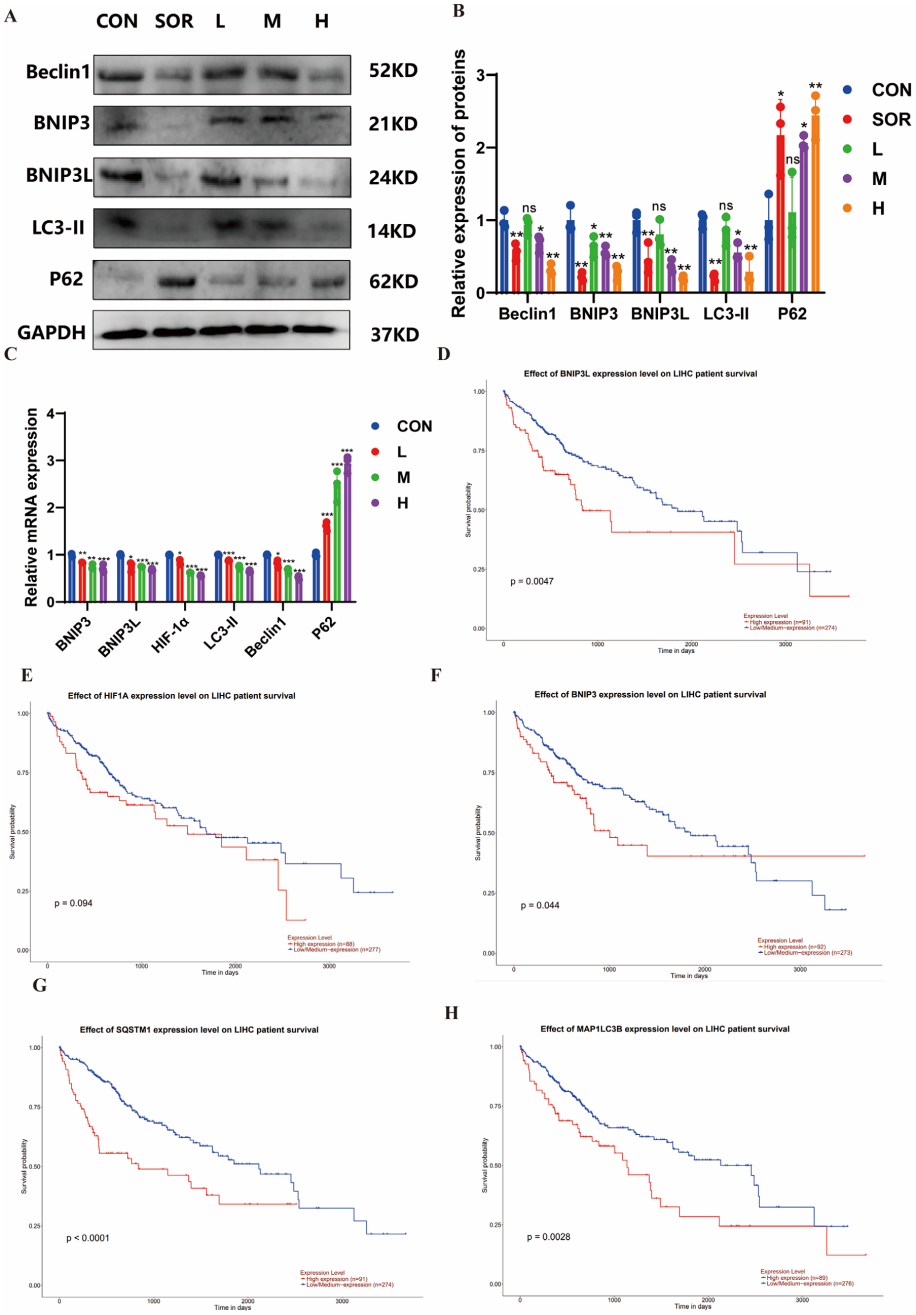
The molecular mechanism of kaempferide to suppress HCC in vivo. (A‐C) Protein expression and gene expression in mice exposed to different concentrations of Kaempferide. (D‐H) Effect of key genes expression level on LIHC patient survival. The data is presented as mean ± standard deviation (*n* = 3). *p < 0.05, **p < 0.01, ***p < 0.001 vs. the CON group.

Aligned with In vitro findings, qRT‐PCR examination of tumour specimens demonstrated diminished expression quantities of BNIP3L, BNIP3, HIF‐1α, Beclin1, BNIP3L, and LC3‐II, coupled with elevated P62 expression in vivo (Figure 7C,p < 0.05). WB analysis confirmed that the protein expression levels aligned with the qRT‐PCR results (Figure 7A,B). The study demonstrated that sorafenib exerts a “pro‐survival” effect in HCC by inducing mitophagy via the PINK/SIAH1 pathway; intervention in this autophagic process significantly enhanced the antitumor efficacy of sorafenib [29]. Zhang et al. reported that sorafenib induces and relies on PINK1‐Parkin‐mediated mitophagy to protect HCC cells, and blocking this autophagy pathway significantly enhances the anti‐tumour effect of sorafenib [30]. To investigate whether sorafenib can inhibit HCC through the BNIP3L‐dependent mitophagy pathway, we performed Western blot analysis on tumour tissues after treatment. The outcomes indicated that the protein expression pattern in the sorafenib cohort mirrored that in the kaempferide cohort, and the precise mechanism requires to be further clarified in subsequent investigations. Ultimately, we used the UALCAN database to investigate the association between the gene expression levels and prognosis in HCC. The expression quantities of BNIP3L, BNIP3 and LC3‐II were discovered to be inversely associated with survival duration (Figure 7D–H). These data strongly demonstrate that kaempferide can improve HCC by downregulating BNIP3L expression to inhibit mitophagy.

## 4 Discussion

The young population is now witnessing a rising incidence of HCC, while optimal curative options are liver resection or liver transplantation (LT). Nonetheless, due to diagnostic delays, most patients are diagnosed at an advanced stage of disease and treated palliatively to ease symptoms and control disease progression without the chance of surgical cure [31]. Research on TCM for HCC has gained increasing attention. Chung et al. reported that TCM‐derived formula AANG modulates Smad3/Smad7 to inhibit multidrug‐resistant HCC progression safely In vitro and in vivo [32]. Galangal has been reported to exert anticancer effects against multiple malignancies. Research has revealed that the pharmacokinetics of kaempferide in galangal are superior to those of kaempferol [22]. Kaempferide imparts anticancer effects on lung cancer cells via ROS‐mediated mechanisms [33]. It can also block the JAK/STAT3 axis to raise ROS levels, thus causing apoptosis of pancreatic cancer cells [34]. Nath et al. reported that kaempferide induces apoptosis in cervical cancer cells, and intraperitoneal toxicity assays in mice verified its safety [35]. Kaempferide has been validated by Martineti et al. to suppress colon cancer cell multiplication and to arrest the cell cycle in the G0/G1 stage [36]. An additional investigation established that kaempferide could diminish Akt phosphorylation quantities and claudin‐2 expression, consequently boosting the sensitivity of lung cancer cells to doxorubicin [37]. The essential consistency of active components between kaempferide in AOE and kaempferide reference standard was first verified in this study by UPLC‐MS/MS (Figure 1). Further CCK8, EdU assays, and flow cytometry experiments showed that kaempferide could inhibit HCC cell proliferation and migration In vitro (Figure 2) and arrest the cell cycle (Figure 4A–D). However, Kaempferide did not induce apoptosis in HCC cells, unlike kaempferol (Figure 4E,F) [11]. Based on MDC method staining assay and transmission electron microscope examination, kaempferide could obstruct the mitophagy pathway in HCC cells (Figure 4G–J, Figure 5, Figure 7). Utilizing the kit for identifying ROS, kaempferide heightened the intracellular quantities of ROS in HCC cells (Figure 5C,D). Furthermore, in vivo experiments verified, that kaempferide could suppress the development of subcutaneous xenograft tumours in nude mice and initially assessed the safety of kaempferide (Figure 6). However, it is yet essential to investigate the molecular mechanism of function of kaempferide targeting HCC.

Through transcriptome‐sequencing, we investigated the differential gene expression between the control and drug‐treated groups and performed GO and KEGG pathway analysis. The results of the sequencing studies revealed that “mitophagy” was common in biological processes and signalling pathways (Figure 3C,D). Notably, BNIP3L was the second most downregulated gene (Figure 3B). Previous studies have demonstrated that in BNIP3L‐dependent mitophagy, the expression levels of HIF‐1α, Beclin1, BNIP3L, and BNIP3 are significantly upregulated [38], which was also validated in this study (Figure 3E,F).

As a mitophagy receptor, BNIP3L binds to Atg8 proteins and recruits autophagosomes to target mitochondria [39]. Hypoxia can induce BNIP3L‐mediated mitophagy, with the core mechanism being that HIF1α can activate the transcriptional expression of BNIP3L [40]. The function of BNIP3L in malignant cells persists contentious, since BNIP3L is regarded as a tumour suppressor gene. It attracts TR3 to mitochondria, inducing autophagic demise in melanoma cells [39]. The RNA oncogene mir‐30d accelerates tumour development by inhibiting BNIP3L expression [41]. Degradation of endogenous BNIP3L enables Ewing sarcoma cell survival [42]. BNIP3L‐facilitated mitophagy might display anti‐cancer impacts via governing immune memory establishment [43]. In contrast, BNIP3L has likewise been acknowledged as an oncogene. Silencing of BNIP3L suppresses mitophagy, thus augmenting fatality in breast, prostate, and renal cancer cells [27]. Investigations have verified that BNIP3L‐triggered mitophagy enhances the persistence of glioblastoma cells, pancreatic cancer cells, and cancer stem cells [44, 45, 46]. BNIP3L‐facilitated mitophagy is autonomous of mitochondrial ubiquitination and can straightforwardly designate autophagosomes as a mitochondrial adapter [47]. This study utilized qRT‐PCR and WB techniques to demonstrate that kaempferide can transcriptionally inhibit the expression of HIF‐1α, BNIP3, BNIP3L, Beclin1, and LC3‐II, while upregulating P62 expression levels. In HCCLM3 cells with BNIP3L overexpression, the expression of its downstream gene LC3‐II was significantly increased, whereas P62 expression was markedly reduced, suggesting that BNIP3L overexpression can activate the mitophagy pathway. Rescue experiment results showed that kaempferide treatment could reverse the above trends (Figure 3E,F, Figure 5). The nude mouse xenograft model validated these In vitro molecular mechanism findings (Figure 7A–C).

Studies have shown that upregulated BNIP3 expression is associated with poor prognosis in patients with primary renal carcinoma and sarcoma [48]. Inhibition of HIF‐1α can suppress the expression of BNIP3 and Beclin‐1, suggesting that the HIF1α/BNIP3/Beclin‐1 axis may constitute a signalling pathway underlying hypoxia‐induced autophagy [49]. Beclin‐1, a protein that regulates autophagy, associates with the anti‐apoptotic protein Bcl‐2 and reduces its affinity for VPS34, thereby inhibiting autophagy [50]. Increased intracellular LC3‐II expression is an indicator of elevated autophagosome formation [51]. The upregulation of P62 expression partially indicates blockage of autophagic flux [52]. Our study demonstrated that kaempferide can suppress the mitophagy pathway and affect HCC cell viability through the downregulation of BNIP3L to regulate the expression quantities of associated mitophagy genes and downstream LC3‐II and P62 (Figure 5 and Figure 7).

Collectively, the outcomes of this investigation propose an initial mechanism whereby kaempferide influences the mitophagy of HCC cells via interacting with BNIP3L, paving the way for exploring other potential mechanisms underlying kaempferide's anti‐HCC effects. These findings may also identify BNIP3L as an intervention target of cellular mitophagy in cancers of multiple cellular origins.

There are still some limitations of this study. According to transcriptome sequencing analysis, kaempferide can also affect HCC through cell cycle regulation, epithelial‐mesenchymal transition, energy metabolism, protein–protein interaction, etc. As findings from our previous study confirmed kaempferide's ability to arrest HCC cells in the G2/M phase via the ATM/CHEK2/KNL1 pathway [11], we did not delve into detail on the mechanism of kaempferide induced cell cycle arrest in HCC. It will be instructive to utilize mouse orthotopic liver xenograft models and primary hepatocellular carcinoma models in future studies to demonstrate the mechanism by which kaempferide inhibits mitophagy in HCC. Likewise, the therapeutic process, adverse effects and prognosis of kaempferide administration need to be evaluated further through extensive clinical trials. The findings of this study enhance the clinical translation of kaempferide as a treatment for HCC.

## Author Contributions

**Lu Lu:** writing – original draft, funding acquisition, visualization, methodology. **Changxian Li:** methodology, data curation. **Cunzhen Jiang:** data curation, visualization. **Yuan Zhao:** visualization, formal analysis. **Yinghong Zhong:** visualization, data curation. **Jicheng Hu:** validation, software. **Zhe Wang:** supervision, software. **Mingyan Zhou:** writing – review and editing, supervision, validation. **Jian Xu:** validation, supervision, funding acquisition. **Xiangcheng Li:** validation, supervision.

## Funding

This study was supported by the National Natural Science Foundation of China (82460875) Hainan Province Science and Technology Special Fund (ZDKJ2021038) Academic Enhancement Support Program of HainanMedical University (XSTS2026196). Joint Program on Health Science & Technology Innovation of Hainan Province (WSJK2026MS242).

## Ethics Statement

All animal experiments were carried out in strict adherence to the ethical and moral guidelines formulated by Hainan Medical University (No. HYLL‐2024‐153).

## Consent

The authors have nothing to report.

## Conflicts of Interest

The authors declare no conflicts of interest.

## Data Availability

The data that support the findings of this study are available from the corresponding author upon reasonable request.
